# Morphoanatomical, Histochemical, and Essential Oil Composition of the *Plectranthus ornatus* Codd. (Lamiaceae)

**DOI:** 10.3390/molecules28186482

**Published:** 2023-09-07

**Authors:** Luiz Renan Ramos da Silva, Zelina Ataíde Correia, Ely Simone Cajueiro Gurgel, Olívia Ribeiro, Sebastião Gomes Silva, Oberdan Oliveira Ferreira, Eloisa Helena de Aguiar Andrade, Mozaniel Santana de Oliveira

**Affiliations:** 1 Postgraduate Program in Biological Biological Sciences–Tropical Botany, Universidade Federal Rural da Amazônia and Museu Paraense Emílio Goeldi, Av. Perimetral, 1901, Terra Firme, Belém 66075-900, Brazil; luizrenan1@hotmail.com (L.R.R.d.S.);; 2 Adolpho Ducke Laboratory—Botany Coordination, Museu Paraense Emílio Goeldi, Av. Perimetral, 1901, Terra Firme, Belém 66077-830, Brazil

**Keywords:** natural products, anatomy, phytochemicals, volatile compounds

## Abstract

*Plectranthus ornatus* is a medicinal and aromatic plant used in traditional and alternative medicine. In this study, leaves of *P. ornatus* were collected in two cities of the state of Pará, “Quatipuru” and “Barcarena”, and were used with the objective of analyzing, through morphoanatomical data and histochemical and phytochemical studies of essential oil, the samples present structural differences and differences in their chemical composition. Anatomical and histochemical analyses were performed by transverse, using longitudinal sections of 8 μm to 10 μm to perform epidermal dissociation, diaphonization, and tests to identify classes of secondary metabolites. The essential oils were isolated by hydrodistillation, and the identification of the chemical composition was performed by gas chromatography coupled with mass spectrometry. The anatomical study shows that there is no difference between specimens collected in different locations, and stellate trichomes were identified. The histochemical study detected total lipids and acids, terpenes, polysaccharides, phenolic compounds, tannins, alkaloids, and calcium oxalate. The low essential oil yield may be related to the low density of secretory cells (glandular trichomes), the unidentified compounds in the highest concentration in the essential oil were in relation to the chemical composition of the essential oils, and the major compounds were α-pinene, sabinene, (*E*)-caryophyllene, caryophyllene oxide, and oct-1-en-3-ol. The results provide new information about the anatomy and histochemistry of *P. ornatus*.

## 1. Introduction

The most famous plants used in traditional medicine are from the *Plectranthus* genus, belonging to the *Lamiaceae* family, including around 200 genus and 3200 vegetable species, and the subfamily *Nepetoideae,* which incorporates plants commonly known as “boldo” [1]. The name *Plectranthus* is derived from the Greek words *plektron* (spur) and *anthos* (flower), describing a stimulus that the flowers have in their support [2]. Many *Plectranthus* species are rich in essential oils, compounds produced in the secondary metabolism of plants. These compounds have demonstrated several applications in the most diverse industrial segments, such as cosmetics, pharmacology, and the food industry [3,4,5], because the complex mixture of the molecules that form essential oils can present potential biological activities. For example, *Plectranthus ornatus* is used in herbal medicine for its potential health benefits [6].

Essential oils (EOs) are formed by complex mixtures of volatile substances. These oils are biosynthesized in the secondary metabolism of plants, and several classes of compounds can be identified in essential oils such as monoterpenes, sesquiterpenes, and phenylpropanoids [7,8,9,10,11]. These complex mixtures is strongly volatile according to abiotic environmental factors. They are colorless to slightly yellow and capable of dissolving in other oils, fats, wax, and ethanol [12]. Their functions include protection, internal and external dispersion against infectious agents on the plant, and attraction of pollinators [13].

Species of the genus *Plectranthus* are characterized by rapid growth and can tolerate a lack of natural resources such as sunlight and rain. In addition, these special species are easy to adapt to the most diverse biomes that can be incorporated, both in Brazil and in other countries [2]. Despise their origin elsewhere, they are notoriously mentioned in Brazil, especially the genus *Plectranthus*. Their diverse use in medicine is reported in ethnobotanic data, and their leaves treat headaches, stomachaches, dermatitis, burns, and allergies to insect bites and act as sanitizers [14].

Moreover, it is difficult to identify the genus *Plectranthus* due to morphological similarities among the species, which contributes to obstacles in defining criteria for taxonomic identification of the genus [15]. Another problem for taxonomic identification is the common names, because in Brazil, for example, there are five different species of the genus *Plectranthus* known by the same common name, “boldo.” Therefore, anatomical and histochemical studies in addition to the chemical composition of essential oils can also be used as a marker of the species or genus *Plectranthus* [16].

It is known that the species *P. ornatus* (*boldo miúdo*, *boldo-chinês*, *falso boldo*) disseminates easily as cultivated or spontaneously, is a remarkable ornament, therapeutic through its secondary metabolites (where diterpenes are the main class of substance), and its morphological aspects may vary according to the habitat where it is placed [10]. However, few studies refer to the anatomical and histochemical approaches of the species *P. ornatus*. In addition, the struggle with properly identifying the species in this genus and their relevance due to medicinal properties might contribute to future surveys from presented Morph anatomical and histochemical data. These plant species are rich in secondary metabolites with applications in various industrial segments [17]; therefore, it is necessary to know the classes of these metabolites and their secretory cells. In this sense, the present work had as its objective to analyze if two samples of *P. ornatus*, collected in different cities of the state of Para-Brazil, have anatomical differences, histochemical differences, and differences in the chemical composition of the essential oils.

## 2. Results and Discussions

### 2.1. Morphological Aspects

**Figure 1:**
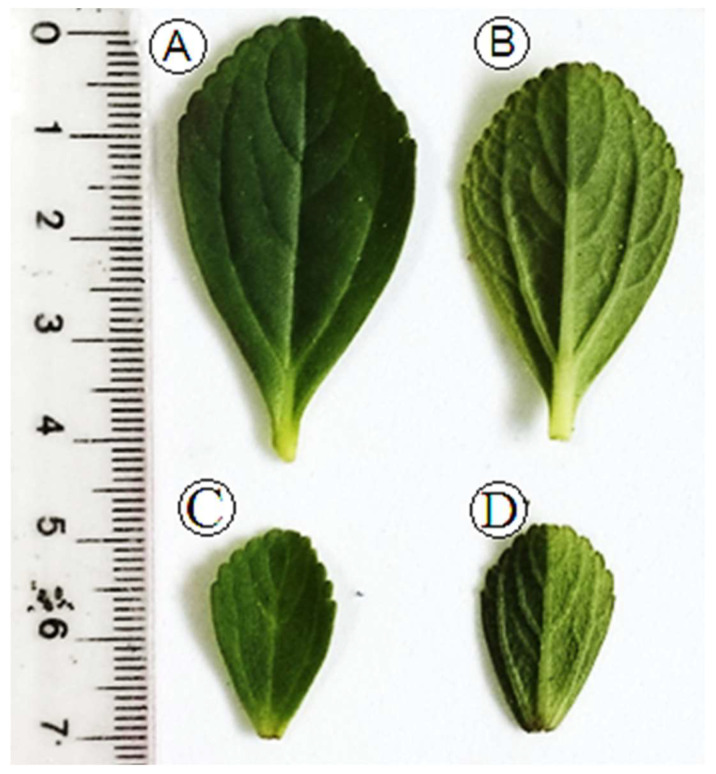
Leaves isolated from *Plectranthus ornatus* boldo. (**A**)—adaxial face from specimen Barcarena. (**B**)—abaxial face from specimen Barcarena. (**C**)—adaxial face from specimen Quatipuru. (**D**)—abaxial face from specimen Quatipuru collected in different sizes throughout the first and fifth internodes.

Morphologically, *P. ornatus* presents with vegetative characteristics similar to other species of the genus *Plectranthus* (Lamiaceae), such as *P. barbatus*, *P. neochilus* [14], *P. amboinicus* [18], *P. verticillatus* [19], and *P. neochilus* [20]. This can also be one of the factors that can hinder the correct taxonomic identification of the species of the genus *Plectranthus*. In the present work, specimens A and B present the same morphological characteristics. The differences were only in the size of the leaves, as can be seen in Figure 1. The leaves presented morphological characteristics of opposite arrangement. They are simple, with an elliptical oval shape, have a dentate margin in the upper half, have small petioles, and are peninerveous. Both the adaxial and abaxial parts have a pilose and succulent surface, that is, they store water and are a kind of mucilage deposit, which can protect the plant from dehydration [21]. This species is characterized by a strong aroma, which can characterize a potential for production and secretion of EOs [22]. Those morphological aspects are confirmed by reported data from the Global Biodiversity Information Facility (GBIF) and *Reflora—Espécies da Flora do Brasil* about *P. ornatus Schltr*.

### 2.2. Anatomical Characterization

**Figure 2:**
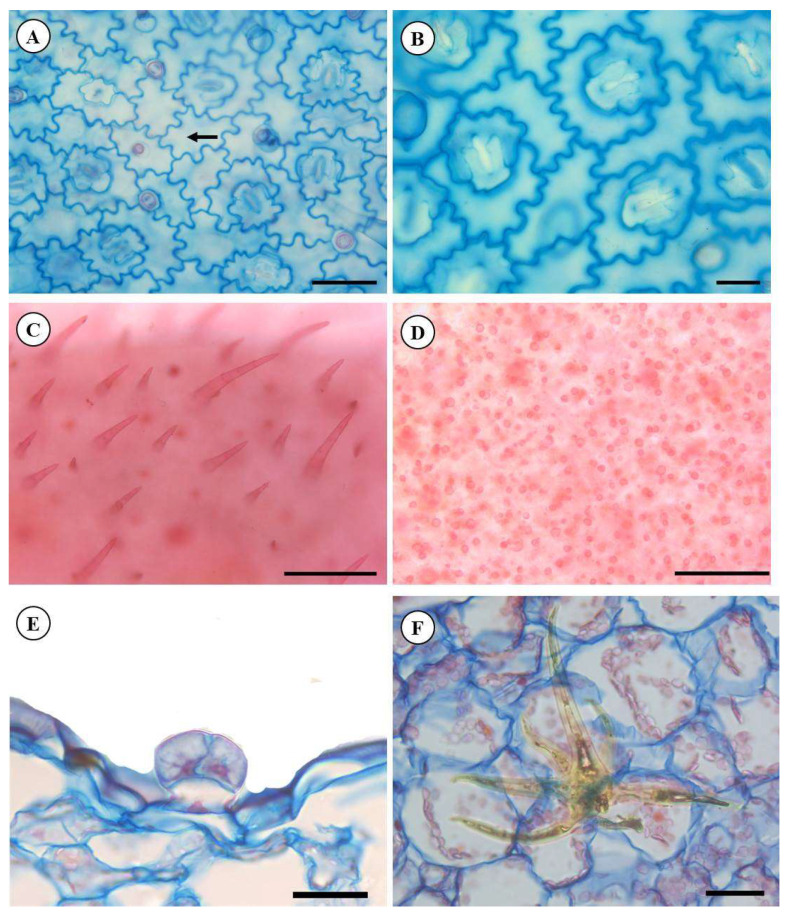
Front view of leaf blade *P. ornatus* Codd. (**A**–**D**). (**A**)—General view of the adaxial side and cells showing sinuous anticlinal walls (arrow). (**B**)—Diacytic stomata detail on the abaxial side. (**C**)—Overview of pluricellular non-glandular trichomes. (**D**)—Overview of glandular trichomes. Cross-sectional area (**E**,**F**). (**E**)—Glandular trichomes with a small petiole. Scale bar: 50 μm (**B**,**E**,**F**), 50 μm (**A**,**C**,**D**).

**Figure 3:**
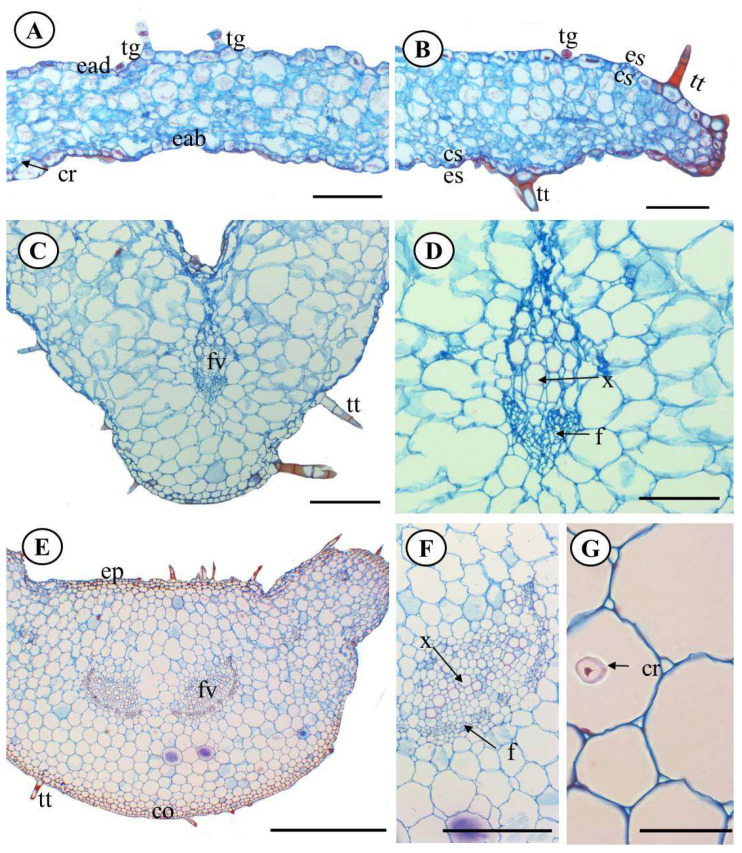
*P. ornatus* Codd. Cross section of the leaf blade. (**A**)—Front view of homogeneous mesophile. (**B**)—Foliar Boldo showing glandular trichome. (**C**)—Midrib. (**D**)—Collateral vascular bundle detail in the midrib. (**E**)—Overview of the petiole. (**F**)—Collateral vascular bundle detail of the petiole. (**G**)—Crystals of calcium oxalate. Legend: ac—angular collenchyma, cr—calcium oxalate crystals, ade—adaxial epidermis, abe—abaxial epidermis, ep—epidermis, st—stomata, sc—substomata camera, vb—vascular bundle, p—phloem, x—xylem, ngt—non-glandular trichome, gt—glandular trichome. Scale bar: 100µm (**A**,**B**,**D**), 200µm (**C**,**F**,**G**), 500µm (**E**).

The anatomical characterization of species *P. ornatus* exhibited that the leaf has in both adaxial and abaxial epidermal sides the presence of epidermal cells and sinuous anticlinal walls (Figure 2A,B). The leaf blade was amphistomatous, and the stomata were diacytic (Figure 3B). Mauro et al. [23], analyzed the anatomical structures of *P. ornatus* and characterized the stomata in a paradermal view, belonging to the anomocytic (irregular-celled) type and presenting reniform guard cells, immersed in epidermal cells with a sinuous contour; however, our results indicate that this classification may not be correct, as the subsidiary cells are organized in such a way that they form a section perpendicular to the stomatal cleft.

Distinct trichomes were found on the two sides of the leaf, being classified as pluricellular uniseriate tectors and glandular as two descriptive characteristics for this species (Figure 2C–E) and representing the main secreting structure found on the leaf of *P. ornatus*. Starred trichomes were also observed (Figure 2F). The trichomes are notable epidermal appendixes for defining the identification within the genus *Plectranthus* [14] for taxonomic and medicinal/pharmacological criteria. As a counterpoint, starred trichomes have not been found in the research of Mauro et al. [23], but these characteristics are proven for the species *P. ornatus* as identified by Ribeiro et al. [24]. In general, the anatomical analyses showed no difference between the specimens studied, as can be seen in Supplementary Material S1.

The cross-sectional area showed that the median leaf region is constituted by uniseriate epidermis and tabular and juxtaposed cells, covered by a smooth and slim cuticle, pluricellular non-glandular trichomes (two to three cells), uniseriate, in a tapered shape and glandular, showing pedicel trichomes, and also sessile glandular trichomes (Figure 3A,B). This morphological trichomes variation on the studied species has been predicted by previous studies [23,24].

A homogeneous mesophile was noted, with no distinction between the palisade and spongy parenchyma and prismatic crystals of calcium oxalate in the idioblasts (Figure 3A). The boundary region was a rounded shape and had compacted cells (Figure 3B).

The cross-sectional area showed that the midrib has a convex format. The epidermis is uniseriate, tabular, and juxtaposed and has a smooth cuticle (Figure 3C). The midrib cortex was formed by 2–3 layers of angular collenchyma next to the epidermis. That is explained by the irregular distance of the cell wall from the sustenance system because it is a mechanical resistance from the vegetative organs still growing [25].

The parenchymal tissue was formed by polygonal, heterodimensional, thin-walled, and slightly sinuous cells. In the central area of parenchymal 1, a collateral vascular bundle was detected (Figure 3D). Those perceptions have also been reported by Mauro et al. (2008) and Ribeiro (2017), as well as for other species belonging to the family, like species *Leonurus sibiricus* L [26].

The petiole on the cross-sectional area had common characteristics with *Peumus boldo, P. neochilus, P. verticillatus,* and *P. amboinicus* [19,23,24,26], such as convex format and projections on the sides. The epidermis was uniseriate and composed of rectangular diminished cells, where both glandular and non-glandular trichomes were present (Figure 3E).

The cells were polygonal, heterodimensional, and thin walled with slight sinuosity (Figure 3E). The vascular bundles were collateral, sclerenchyma-closed, surrounding the phloem, and two secondary bundles were also present in the sideways (Figure 3F). Crystals of calcium oxalate in the idioblasts were also noted (Figure 3G).

When the xylem and phloem are placed on opposite sides, as labeled in the anatomy of the species, the classification is “collateral” as predicted by the guidelines of morphological and anatomical classification of [27].

The presence of calcium oxalate, which was more concentrated in the petiole, reflects an adaptation in plants derived from differences in external factors, such as herbivory differences and formation of vegetative structures, and turns them into toxic ones [28], as observed in *Plectranthus neochilus* Schltr and reported by Duarte et al. [26].

### 2.3. Histochemistry

**Figure 4:**
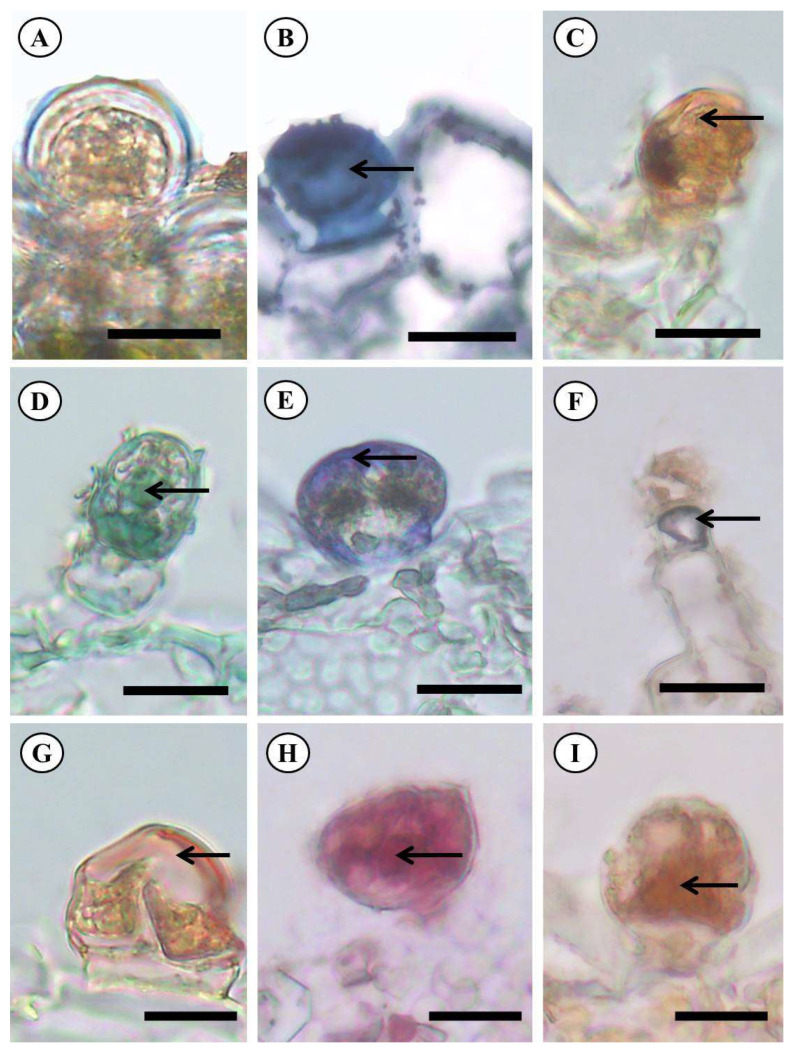
Histochemical tests on the leaf limbo secreting glandular trichomes of *Plectranthus ornatus* Codd. (**A**)—White, (**B**)—Positive result for total lipids with Sudan Black. (**C**)—Positive result for total lipids with Sudan III. (**D**)—Positive result for acidic lipids. (**E**)—Positive result for terpenes. (**F**)—Positive result for total phenolic compounds. (**G**)—Positive result for tannin. (**H**)—Positive result for total polysaccharides. (**I)**—Positive result for alkaloids. Arrows: Indicate where the reactions happened. Scale bar: 20 μm (**G**–**I**) 50 μm (**A**–**F**).

Histochemical tests suggested in this survey that the margin and petiole of *P. ornatus* reacted for distinct classes of secondary metabolites in glandular trichomes. According to Kalicharan et al. [29], global characterization detection usually happens in the nucleus of glandular specialized cells in trichomes, and the reagents react more evidently when metabolites are identified on glandular secretions. This is derived from the ability of glandular trichomes to store and secrete an amount of secondary metabolites [30]. The results of histochemical analysis are reported in Table 1, and the reactions are expressed in (Figure 4A–I).

The trichome secretions of *Plectranthus ornatus* reacted (positive result) for total lipids and acidic lipids. However, there is a difference in lipidic composition, as evidenced by the Nadi reagent. This reaction stained purplish, indicating terpenes of different sizes (molecular masses), implicating a mixture of essential oil and resin [31]. The terpene identification is more effective when the reaction happens (positive result), as seen in this study and also in previous works with *P. amboinicus,* Mentha x villosa Huds [18,32]. Genus *Ocimum* terpenes can also be found in the stem [33].

Those oil compositions might influence the ecological relationship established between the plant and the environment [18]. Hence, environmental conditions/disturbances are also a strong influencing factor on the yield of other substances and chemical compounds such as EOs, because their production is related to the leaf’s protection against external factors such as herbivory factors. Their production also helps pollination and aids in herbal action on the human body when using traditional medicine, as it acts as an analgesic, an antibacterial, and an antioxidant [13,34].

The tests on the species showed the presence of phenolic compounds and tannin, which has also been found in other species belonging to the family *Lamiaceae*, along the leaves of *Plectrathus amboinicus* (Lour.) Spreng [18] and every vegetative part but the inflorescence of *Ocimum basilicum* and *O. campechianum* [32]. The work of Rocha et al. [33] confirmed the positive result for tannin in histochemical tests, potentializing the probable explanation of the resistance and binder power of this plant.

Phenolic compounds are widely found substances in vegetables according to Silva et al. [35]. This functional group highlights substances such as coumarins, lignin, quinones, tannins, and flavonoids [36]. Phenolic compounds in vegetables might act against herbivores and pathogens, promote cell reconstruction, and attract pollinizers [37]. They also have healing, antioxidant, anti-inflammatory, and antimicrobial action [38].

Glandular trichomes from the analyzed species were demonstrated to have alkaloids likewise observed in other literature [18,39], on the leaves of *Plectrathus amboinicus* (Lour.) Spreng, and by Alasbahi, and Melzig [40], on leaves of *Plectranthus barbatus* Andrews. The alkaloids are some of the most important nitrogenated compounds found in herbal vascular plants, providing herbivory protection, along with anesthetic, antitumoral, myorelaxant, and antimicrobial activities [41].

The positive result for periodic acid-Schiff (PAS) pointed to the presence of total polysaccharides in glandular trichomes of *P. ornatus*. The polysaccharides are stocking molecules of hydrophilic carbohydrates that function in cell wall formation, and by their chemical properties, they prevent damages caused by hydric stress [42,43]. On the other hand, the polysaccharides from exudates are produced as a defense mechanism against physical injuries and microbial attack [44]. Therefore, the most relevant secondary metabolites found in the secretion of glandular trichomes of *P. ornatus* have defensive action, which somehow helps with survival in the environment.

### 2.4. Chemical Composition of the Essential Oil

The essential oils of specimens (A) and (B), as shown in Table 2, did not yield properly (0.1 mL), but the chemical characterization was still performed. A total of 93.45% and 99.92%, respectively, of the 93 compounds were identified and are described in Table 3. The hydrocarbon sesquiterpenes play a role of 27.56% in the chemical profile of essential oil from specimen (A) collected in the city of Barcarena, Brazil, and 41.65% for (B) collected in the city of “Quatipuru”, Brazil. Sesquiterpenes were also detected in essential oil (EO) from other species of the genus *Plectranthus*. However, an amount of 63.1% of monoterpenes was identified in a sample of *P. ornatus* and was identified in a sample collected by other authors [45] in the state of Ceara, Brazil.

The major components in the essential oil of specimen (A) were (*E*)-Caryophyllene (12.84%), α-pinene (12.38%), and sabinene (8.72%). On the other hand, the EO from specimen (B) was found to have (*E*)-caryophyllene (29.61%), 1-octen-3-ol (13.92%), and terpinen-4-ol (5.66%). The results varied from what has been found in a sample of *P. ornatus* collected in Lisboa, Portugal, in which the predominant substances were oct-1-en-3-ol (13–31%), β-pinene (11–24%), α-pinene (11–19%), and β-caryophyllene (11%) [55].

Literature reports the presence of monoterpenes in essential oils from genus *Plectranthus* as observed in EO from *P. amboinicus*, which showed in its chemical profile the following substances: carvacrol (23.0%), camphor (22.2%), δ-3-carene (15.0%), γ-terpinene (8.4%), *o*-cymene (7.7%), and α-terpinene (4.8%) [56]. (*E*)-caryophyllene was also observed in other genus studies such as in *P. grandis*, which had a content of (*E*)-caryophyllene (38.25%) higher than that found in the present work. In addition, *P. grandis* EO exhibited sesquiterpene compounds such as α-copaene (13.23%) and germacrene (11.38%). In *P. ornatus* EO, the major chemical components were caryophyllene oxide (61.74%) and β-caryophyllene (10.65%) [57].

In the work of Passinho-Soares et al. [58], a sample of *P. ornatus* was collected from a parent plant (main plant) cultivated at the Pharmacy College of Federal University of Bahia (*Universidade Federal da Bahia, UFBA*), and the major components of its EO were α-terpinyl acetate and the monoterpenes (α-thujene, α-pinene, β-pinene, camphene, sabinene, and α-limonene). Another study also detected monoterpenes, such as α-thujene (4.40%), β-bourbonene (8.07%), and 1-octen-3-ol (11.85%), in the EO of *P. ornatus* [59].

## 3. Materials and Methods

### 3.1. Collecting Area for the Botanical Material

The botanical material was collected in two different cities in the state of Para, Brazil. Sample A was collected in a garden of a local producer in the city of Barcarena (state of Para) at the following geographical coordinate: latitude 01°30′21″ south and longitude 48°37′33″ west. Sample B was collected in the city of Qautipurú (state of Para) located at the geographical latitude 0°53′56″ south and longitude 47°0′40″ west. Samples of specimens of *P. ornatus* were identified and deposited in the Herbarium of *Museu Paraense Emilio Goeldi*, João Murça Pires, registered with number MG N° 245302.

### 3.2. Morphological Analysis

The morphological leaf parameters were analyzed under the eyes of taxonomic reviews [60] and specialized literature [61] about leaf morphology to characterize and analyze the vegetative parts such as apex, margin, foliage, midrib, phyllotaxy, and petiole using magnifying glasses. The aerial vegetative parts of the species were collected for anatomical and histochemical studies, developed in the Vegetable Anatomy Laboratory (*Laboratório de Anatomia Vegetal*), in the Botanic Coordination of *Museu Paraense Emílio Goeldi* Museum—LAVEG/COBOT/MPEG, and have undergone the following techniques.

### 3.3. Anatomical Analysis of the Leaf Blade by Light Microscopy (LM)

**Figure 5:**
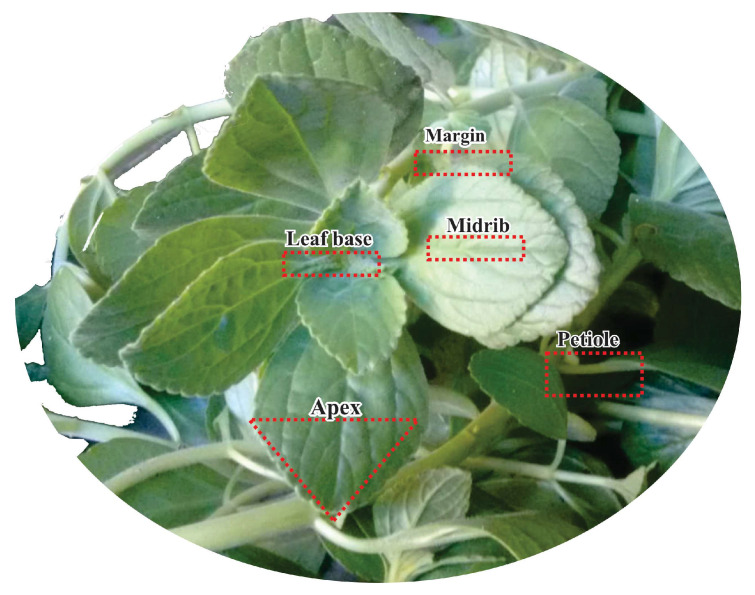
Regions of the leaf blade of *Plectranthus ornatus* used in the anatomical study.

The samples (Figure 1) of the leaf blade were sectioned into the regions used in the anatomical study (margin, midrib, and petiole), fixed on FAE (formaldehyde, acetic acid, and ethanol 50%) [47] for 24 h for the structural study, in hydrophilic substances for histochemical tests, in NBF (neutral-buffered formalin) for 48 h for lipophilic substances [62] and stored in ethanol 70%. Figure 5 displays the image of the leaf sections of *Plectranthus ornatus*.

For laboratory analysis, the samples previously stored in ethanol 70% were submitted for dehydration in a growing butyric alcohol series (butyric tertiary alcohol) to include it in histological paraffin with DMSO (Paraplast^®^, ©Leica Biosystems, Teaneck, NJ, USA) [47]. Transversal and longitudinal sections from 8 μm to 10 μm were obtained with the aid of a Rotary Microtome (model Leica^®^ RM 2245, Leica^®^ Biosystems, Heidelberg, Alemanha, Germany). One of the samples was stained with Astra Blue and Safranine (Gerlach, 1969), and the other was destined for histochemical tests. The blades were prepared in colorless Canada balsam [63].

### 3.4. Epidermis Dissociation

For epidermis dissociation, smaller fractions of leaf were sectioned with the aid of a steel blade and immersed in hydrogen peroxide and acetic acid solution for 24 h [64], washed with distilled water, stained with Astra Blue and Safranine [65], and placed in aqueous glycerin (1:1). Their edges were insulated using colorless enamel, by Purvis et al. [66].

### 3.5. Diaphanization

The *in natura* leaves were dehydrated in ethanol decreasing series (50%, 30%, and 10%) for 30 min, washed with distilled water, and placed on sodium hydroxide 5% for 2 h. Then, the samples were placed on sodium hypochlorite 20% until clarification, when they were washed with distilled water for 30 min and dehydrated in ethylic growing series (until ethanol was at 50%), stained with Safranine + ethanol 50% [67,68], and placed on the synthetic resin [69].

### 3.6. Histochemistry

Histochemical tests were performed on sectioned samples that were handmade and placed in histological paraffin with DMSO (Paraplast^®^, ©Leica Biosystems, Richmond, IL, USA) [47]. Table 3 shows the kinds of reactions carried out for detecting the major groups of metabolites.

The control samples were studied according to the corresponding techniques from the author’s tests. The photomicrographs were done on a microscope (Leica DM6B, Wetzlar, Germany W) attached to a digital camera (Leica application suite LAS V4. 12) at the Microscopy Laboratory in *Paraense Emílio Goeldi* Museum (MPEG), calibrated with suitable micrometer blades as specified by the manufacturer.

### 3.7. Essential Oil Extraction by Hydrodistillation

For essential oil extraction, 90 g of the botanic *Plectranthus ornatus* was dried in a drying room where air humidifiers were used. Then, it was extracted by hydrodistillation, using a Clevenger-type glass system modified and attached to a cooling system for maintaining the condensation water between 10–15 °C for 3 h, as described in Franco et al. [70] and de Oliveira et al. [71].

### 3.8. Chemical Composition Analysis

The chemical composition of essential oils was evaluated by gas chromatography-mass spectrometry (GC/MS), using a Shimadzu QP-2010 plus system with the subsequent conditions: silica capillary column Rtx-5MS (30 m × 0.25 mm, film thickness 0.25 μm); programmed temperature (60–240) °C to 3 °C/min; injector temperature of 250 °C; helium as carrying gas (linear velocity of 32 cm/s, measured at 100 °C); and injection without division (1 μL of a hexane 2:1000 solution). The ionization was performed by the electron impact technique (70 eV), and the ion source temperature, along with the other parts, was fixed to 200 °C. The quantification of volatile compounds was determined by gas chromatography with a flame ionization detector (FID; Shimadzu, Kyoto, Japan, system QP 2010) at the very same conditions as expressed for GC/MS, except for the carrying gas, which was hydrogen. The retention index was calculated for every volatile constituent eluting a homologous series of n-alkanes (C_8_–C_40_), and the spectra obtained experimentally were compared with literature, alongside the retention indexes [46,72,73].

## 4. Conclusions

According to the evaluated analysis, despite the plants being located in different places and of different sizes, they have proven to be from the same species *P. ornatus*, showing the same anatomical and histochemical characteristics in relation to the chemical composition of the isolated essential oils. The differences were more in quantitative levels than in qualitative terms. Sample (A) was identified as having α-pinene, sabinene, (*E*)-caryophyllene, and caryophyllene oxide as the majority, while sample (B) had oct-1-en-3-ol, (*E*)-Caryophyllene, and caryophyllene oxide, which may be related to the biome in which the specimens were inserted. The low yield of essential oils in both samples may also be related to the production of resin oil in their secretory glands, which may enhance production of calcium oxalate, as this substance is toxic and can serve as a defense mechanism to protect the species.

## Figures and Tables

**Table 1:** Results of histochemistry applied on the leaf of *P. ornatus*, samples (A) and (B).

Test	Secretory Structures	
	Glandular Trichomes	Idioblasts
Total lipids	+	*n*
Acidic lipids	+	*n*
Terpenes	+	*n*
Total polysaccharides	+	*n*
Starch	−	*n*
Phenolic compounds	+	*n*
Tannin	+	*n*
Alkaloids	+	*n*
Crystals of calcium oxalate	*n*	+

Note: (*n*) not applied, (+) positive result, (−) negative result.

**Table 2:** Chemical composition of essential oil isolated from two specimens of *P. ornatus*, (**A**) collected in the city of “Barcarena”-Brazil and (**B**) collected in the city of “Quatipuru”-Brazil.

IR_C_	IR_L_	Compounds	(A)	(B)
919	924	α-Thujene	2.82	0.22
935	932	*α*-Pinene	12.38	
969	969	Sabinene	8.72	
970	974	Oct-1-en-3-ol		13.92
986	988	Octan-3-ol	1.06	4.04
999	1001	Hex-(3*E*)-enyl acetate	0.8	
1011	1014	α-Terpinene	1.16	0.6
1018	1020	*p*-Cymene	4.08	
1022	1022	*o*-cymene	3.64	2.74
1040	1044	(*E*)-β -Ocimene	0.5	1.03
1051	1054	γ-Terpinene	2.66	2.19
1060	1065	*cis*-Sabinene hydrate (IPP vs. OH)		0.4
1082	1085	Terpinolene	0.95	0.7
1093	1095	Linalool	0.2	1.23
1097	1098	Linalool		0.24
1111	1112	*trans*-Thujone	0.59	0.33
1115	1119	α-*p-*Mentha-2,8-dien-1-ol		0.45
1120	1122	α-Campholenal	0.77	0.37
1133	1135	(*E*)-Pinocarveol	0.51	
1124	1122	α-Campholenal		0.06
1134	1139	*trans*-Pinocarveol		0.86
1139	1137	*trans*-Sabinol (trans for OH vs. IPP)		0.14
1157	1160	Pinocarvone	0.28	0.15
1160	1166	*p*-Mentha-1,5-dien-8-ol	1.23	0.58
1171	1174	Terpinen-4-ol	2.9	5.66
1178	1181	Thuj-3-en-10-al	0.21	
1185	1186	α-Terpineol	0.49	0.84
1191	1194	Myrtenol		0.49
1192	1195	Methyl chavicol	1.13	
1199	1201	*n*-Decanal	0.16	
1246	1235	*trans*-Chrysanthenyl acetate	0.16	
1280	1283	Bornyl acetate	0.7	
1344	1346	α-terpinyl acetate	4.1	
1346	1348	α-Cubebene		0.99
1372	1374	α-Copaene	1.11	0.69
1381	1387	β-Bourbonene	2.54	1.18
1386	1387	β -Cubebene	0.61	0.74
1388	1389	β -Elemene	0.12	
1389	1390	Sativene	0.1	
1417	1417	(*E*)-Caryophyllene	12.84	29.61
1425	1430	β -Copaene	0.41	0.3
1431	1432	*trans*-α-Bergamotene	0.16	
1440	1447	Isogermacrene-D	0.13	
1441	1440	(*Z*)-β -Farnesene		0.09
1446	1448	*trans*-Muurola-3,5-diene	0.13	0.06
1450	1452	α-Humulene	1.03	1.22
1457	1458	*allo*-Aromadendrene	0.03	
1463	1460	dehydro Aromadendrane	0.05	
1470	1471	Dauca-5,8-diene	0.16	
1472	1478	γ-Muurolene	0.14	0.16
1474	1481	γ-Curcumene	0.05	
1482	1484	Germacrene-D	1.53	2.25
1489	1493	*trans*-Muurola-4(14),5-diene		0.11
1491	1495	γ-Amorphene	0.22	
1492	1498	ε-Amorphene		0.42
1497	1500	α-Muurolene	0.14	
1504	1505	α-Bulnesene	3.39	1.65
1507	1513	γ-Cadinene	0.34	0.26
1510	1514	β -Curcumene	0.14	
1512	1513	γ-Cadinene		0.57
1511	1514	Cubebol	0.23	
1522	1522	δ-Cadinene	2.07	1.35
1524	1529	Kessane	1.04	
1528	1533	*trans*-Cadina-1,4-diene	0.12	
1539	1542	*trans*-Sesquisabinene hydrate	0.16	
1549	1547	Italicene epoxide	0.4	0.31
1558	1561	(*E*)-Nerolidol	0.35	0.2
1575	1577	Spathulenol	0.08	
1583	1582	Caryophyllene oxide	9.62	11.76
1595	1586	Thujopsan-2-α-ol	0.05	
1598	1600	Cedrol	0.12	
1606	1608	Humulene epoxide II	0.74	0.55
1612	1618	1,10-di-epi-Cubenol	0.04	
1625	1627	1-epi-Cubenol	0.27	0.18
1630	1631	*trans*-Sesquilavandulol		0.05
1629	1639	allo-Aromadendrene epoxide-	0.25	
1633	1639	Caryophylla-4(12),8(13)-dien-5-α-ol	0.5	0.31
1637	1644	α-Muurolol	0.72	0.76
1642	1645	Cubenol	0.08	0.06
1651	1652	α-Cadinol	0.32	0.4
1662	1661	Allohimachalol	0.21	
1655	1666	14-hydroxy-(*Z*)-Caryophyllene		0.26
1668	1668	14-hydroxy-9-epi-(*E*)-Caryophyllene	1.77	1.01
1675	1679	Khusinol	0.16	
1683	1685	Germacra-4(15),5,10(14)-trien-1- α-ol	0.33	0.18
1743	1746	8- α-11-Elemodiol	0.06	
1786	1792	Drimenone	0.1	
1837	1841 *	Phytone	0.11	0.2
2092		9-Octadecenoic acid (Z)-, methyl ester	1.37	
2105	2095	Methyl linoleate	0.61	
2107		Phytol derivative		2.49
2118	2124	Methyl octadecanoate	0.09	
2213	2218	e-Phytol acetate		2.31
2215		Terpenoids not identified	4.04	22.96
		Hydrocarbon Monoterpenes	36.91	7.48
		Oxygenated Monoterpenes	7.34	11.8
		Hydrocarbon Sesquiterpenes	27.56	41.65
		Oxygenated Sesquiterpenes	17.6	16.03
		Terpenoids not identified	4.04	22.96
		Total	93.45	99.92

RIC = Calculated retention index; RIL = Literature retention index. * Mondello [46].

**Table 3:** Histochemical tests applied to the secretory structures of *Plectranthus ornatus* Codd. leaf for detecting its major classes of metabolites.

Metabolic Groups	Reagents	Reaction
Total lipids	Sudan III (Johansen [47])	Orange
	Sudan Black (Pearse [48])	Blue
Acidic lipids	Nile blue sulfate (Cain [49])	Greenish blue
Terpenes	Nadi reagent (David; Carde [50])	Purple
Total Polysaccharides	PAS (Periodic acid Schiff) (MCmanus [51])	Pink
Tannin	Chloridric Vanilla (Mace; Howell [52])	Reddish orange
Starch	Lugol’s solution (Johansen [47])	No reaction
Phenolic compounds	Iron(III) chloride 10% (Johansen [47]).	Black
Alkaloids	Dragendorf reagent (Svendsen; Verpoorte [53])	Reddish brown
Crystals of calcium oxalate	Hydrochloric acid 5% (Chamberlain [54])	Until the dissociation of crystals

## Data Availability

Not applicable.

## Supplementary Materials

The following supporting information can be downloaded at https://www.mdpi.com/article/10.3390/molecules28186482/s1: Species figures (A) and (B) show no anatomical difference.

## Sample Availability

Samples of the compounds in *Museu Paraense Emílio* Goeldi are available from the authors.
