# Supplementary material for: Morphoanatomical, Histochemical, and Essential Oil Composition of the Plectranthus ornatus Codd. (Lamiaceae)
Source: Molecules. 2023 Sep 7;28(18):6482. doi: 10.3390/molecules28186482 (PMC10536712; doi:10.3390/molecules28186482)
Supplement: Supplementary file 1 [file molecules-28-06482-s001.zip › molecules-2568008-supplementary.pdf]

Supplementary Material

Morphoanatomical, Histochemical, and Essential Oil Composition of the *Plectranthus ornatus* Codd. (Lamiaceae)

Luiz Renan Ramos da Silva [1], Zelina Ataíde Correia [1], Ely Simone Cajueiro Gurgel [1], Olívia Ribeiro [1], Sebastião Gomes Silva [2], Oberdan Oliveira Ferreira [2], Eloisa Helena de Aguiar Andrade [1,2] and Mozaniel Santana de Oliveira [1,2,*]

[1] Postgraduate Program in Biological Biological Sciences–Tropical Botany, Universidade Federal Rural da Amazônia and Museu Paraense Emílio Goeldi, Av. Perimetral, 1901, Terra Firme, Belém 66075-900, Brazil; luizrenan1@hotmail.com (L.R.R.d.S.)

[2] Adolpho Ducke Laboratory—Botany Coordination, Museu Paraense Emílio Goeldi, Av. Perimetral, 1901, Terra Firme, Belém 66077-830, Brazil

* Correspondence: mozaniel.oliveira@yahoo.com.br

Abstract: *Plectranthus ornatus* is a medicinal and aromatic plant used in traditional and alternative medicine. In this study, leaves of *P. ornatus* were collected in two cities of the state of Pará, "Quatipuru" and "Barcarena", were used with the objective of analyzing, through morphoanatomical data, histochemical, and phytochemical studies of essential oil, the samples present structural differences and in their chemical composition. Anatomical and histochemical analyzes were performed by transverse, and longitudinal sections of 8µm to 10µm to perform epidermal dissociation, diaphonization, and tests to identify classes of secondary metabolites. The essential oils were isolated by hydrodistillation and the identification of the chemical composition was performed by gas chromatography coupled to mass spectrometry. The anatomical study shows that there is no difference between specimens collected in different locations, stellate trichomes were identified. The histochemical study detected total lipids and acids, terpenes, polysaccharides, phenolic compounds, tannins, alkaloids, and calcium oxalate. The low essential oil yield may be related to the low density of secretory cells (glandular trichomes), the unidentified compounds in the highest concentration in the essential oil were in relation to the chemical composition of the essential oils the major compounds were α-pinene, sabinene, (*E*)-caryophyllene, caryophyllene oxide, and oct-1-en-3-ol. The results provide new information about the anatomy and histochemistry of *P. ornatus*.

Keywords: Natural products; anatomy; phytochemicals; volatile compounds

---

Supplementary Material S1

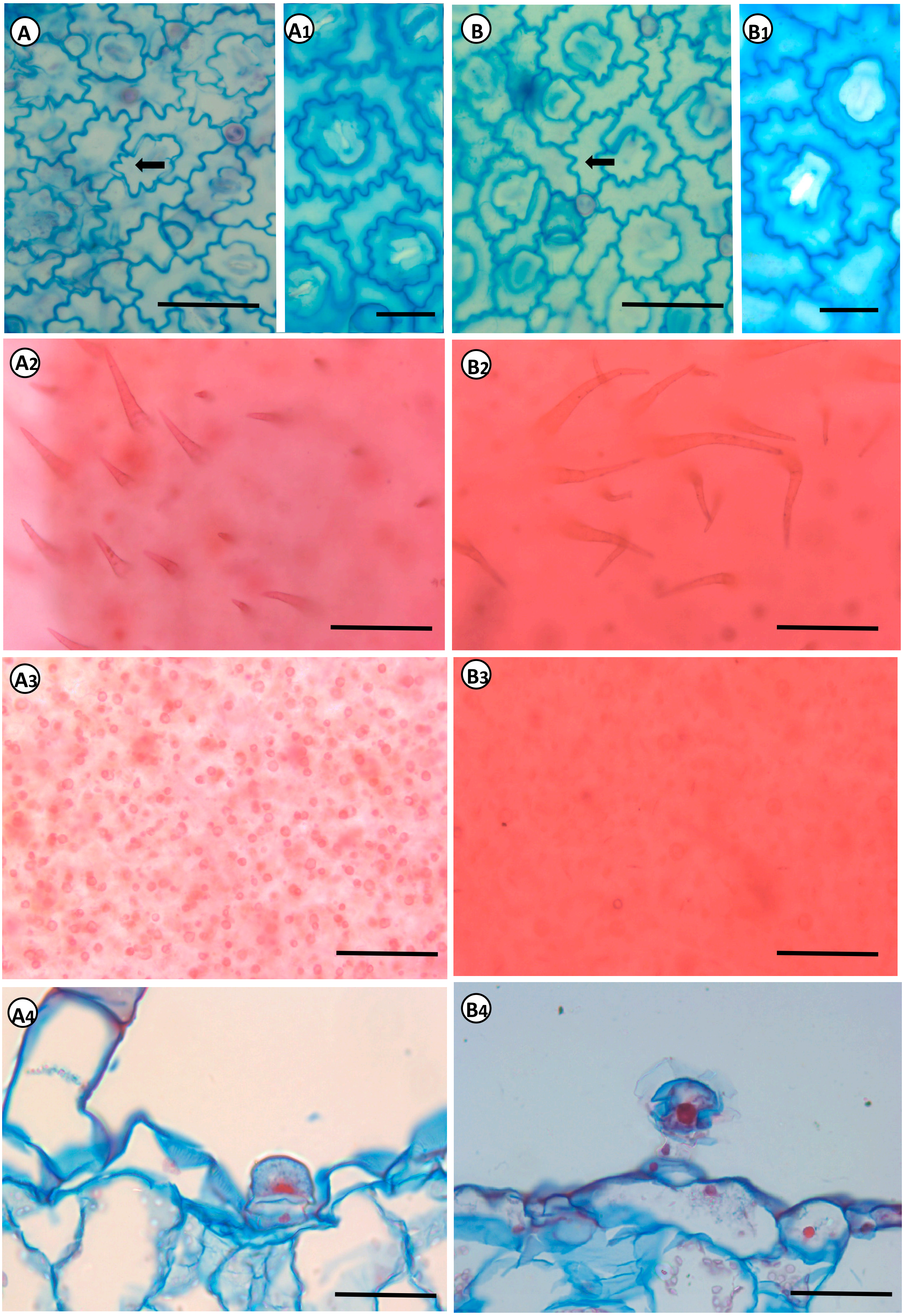
A
A1
B
B1
A2
B2
A3
B3
A4
B4

Figure 1. Front view of the leaf blade of specimens of *Plectranthus ornatus* Codd, being from Barcarena represented by the letter A (A-A1,A2,A3,A4) and the specimen from Quatipuru represented by the letter B (B-B1,B2,B3,B4) . A and B- General view of the adaxial face and cells with sinuous anticlinal walls (arrow), A1 and B1- Detail of the diacytic stomata on the abaxial face, A2 and B2- General view of the tector unicellular pluricellular trichomes, A3 and B3- General view of the glandular trichomes. In cross section. A4 and B4- Glandular trichome. Scale bar: 50µm (A-A1,A2,A3,A4) 50µm (B-B1,B2,B3,B4).

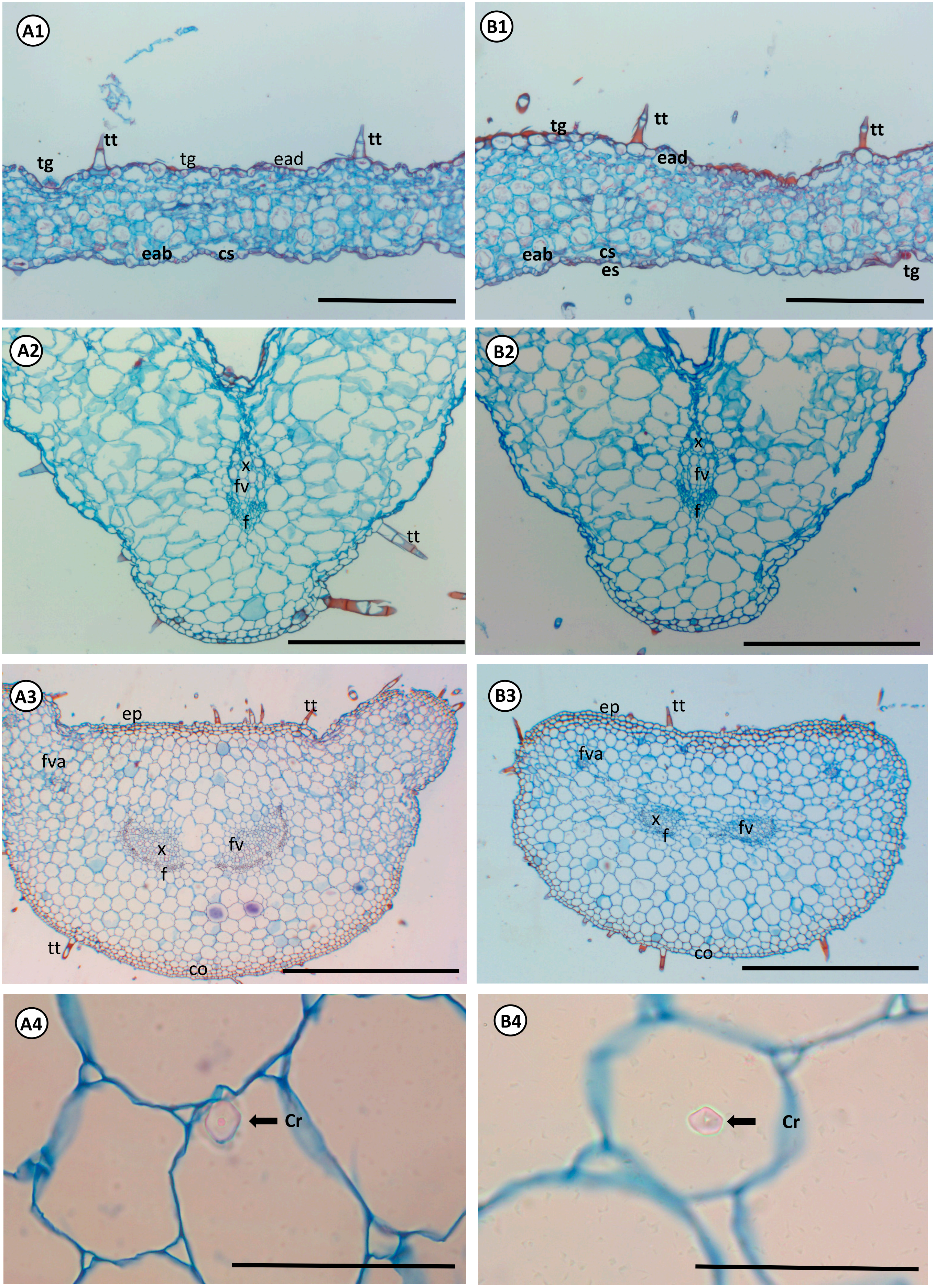
A1
tt
tg
tg
ead
tt
eab
cs
B1
tg
tt
tt
ead
eab
cs
es
tg
A2
x
fv
f
tt
B2
x
fv
f
A3
ep
tt
fva
x
fv
f
tt
co
B3
ep
tt
fva
x
f
fv
co
A4
Cr
B4
Cr

Figure 2. Specimens of *Plectranthus ornatus* Codd. Cross section of leaf blade. Being A (A1,A2,A3,A4) the specimen from Barcarena, and being B (B1,B2,B3,B4) the specimen from Quatipuru. A1 and B1- General view of the homogeneous mesophyll with different trichomes, A2 and B2- Midrib focusing on the collateral vascular bundle of the midrib, A3 and B3- General view of the petiole with the collateral vascular bundle of the petiole, A4 and B4- Crystal of calcium oxalate. Caption: angular collenchyma, cr- calcium oxalate crystals, ead- adaxial epidermis, eab- abaxial epidermis, ep- epidermis, stomata, cs- substomatic chamber, fv- vascular bundle, f- phloem, x- xylem , tt- tector trichome, tg- glandular trichome. Scale bar: 100µm (A1, B1), 200µm (A2,B2,A3,B3), 500µm (A4,B4).
